# Multi-Trait Genomic Risk Stratification for Type 2 Diabetes

**DOI:** 10.3389/fmed.2021.711208

**Published:** 2021-09-08

**Authors:** Palle Duun Rohde, Mette Nyegaard, Mads Kjolby, Peter Sørensen

**Affiliations:** ^1^Department of Chemistry and Bioscience, Aalborg University, Aalborg, Denmark; ^2^Department of Health Science and Technology, Aalborg University, Aalborg, Denmark; ^3^Department of Biomedicine, Aarhus University, Aarhus, Denmark; ^4^Department of Population Health and Genomics, University of Dundee, Dundee, United Kingdom; ^5^Department of Clinical Pharmacology, Aarhus University Hospital, Aarhus, Denmark; ^6^Steno Diabetes Center Aarhus, Aarhus University Hospital, Aarhus, Denmark; ^7^Centre for Quantitative Genetics and Genomics, Aarhus University, Aarhus, Denmark

**Keywords:** UK Biobank, genetic risk scores, GRS, multi-trait analysis, precision medicine

## Abstract

Type 2 diabetes mellitus (T2DM) is continuously rising with more disease cases every year. T2DM is a chronic disease with many severe comorbidities and therefore remains a burden for the patient and the society. Disease prevention, early diagnosis, and stratified treatment are important elements in slowing down the increase in diabetes prevalence. T2DM has a substantial genetic component with an estimated heritability of 40–70%, and more than 500 genetic loci have been associated with T2DM. Because of the intrinsic genetic basis of T2DM, one tool for risk assessment is genome-wide genetic risk scores (GRS). Current GRS only account for a small proportion of the T2DM risk; thus, better methods are warranted for more accurate risk assessment. T2DM is correlated with several other diseases and complex traits, and incorporating this information by adjusting effect size of the included markers could improve risk prediction. The aim of this study was to develop multi-trait (MT)-GRS leveraging correlated information. We used phenotype and genotype information from the UK Biobank, and summary statistics from two independent T2DM studies. Marker effects for T2DM and seven correlated traits, namely, height, body mass index, pulse rate, diastolic and systolic blood pressure, smoking status, and information on current medication use, were estimated (i.e., by logistic and linear regression) within the UK Biobank. These summary statistics, together with the two independent training summary statistics, were incorporated into the MT-GRS prediction in different combinations. The prediction accuracy of the MT-GRS was improved by 12.5% compared to the single-trait GRS. Testing the MT-GRS strategy in two independent T2DM studies resulted in an elevated accuracy by 50–94%. Finally, combining the seven information traits with the two independent T2DM studies further increased the prediction accuracy by 34%. Across comparisons, body mass index and current medication use were the two traits that displayed the largest weights in construction of the MT-GRS. These results explicitly demonstrate the added benefit of leveraging correlated information when constructing genetic scores. In conclusion, constructing GRS not only based on the disease itself but incorporating genomic information from other correlated traits as well is strongly advisable for obtaining improved individual risk stratification.

## Introduction

Type 2 diabetes mellitus (T2DM) is a chronic disease with severe comorbidities, such as myocardial infarction, loss of kidney function, blindness, and risk of amputations (1). Globally, the prevalence of T2DM is expected to increase exponentially in developing countries (2, 3), and it is a disease that places a severe economic burden on health systems. Accurate disease risk assessment is important for early disease diagnosis for initiating lifestyle changes early in the disease progression or prompt the clinician to treat high-risk patients more aggressively, which is expected to slow down disease progression, reduce disease symptoms, and prevent severe morbidity and mortality. Thus, methods for accurate disease risk assessment are absolutely critical for reducing morbidity and mortality.

Studies have unambiguously shown that T2DM is a complex, multifactorial disease, where an individual's risk of developing the disease is influenced by a combination of genetic variation at multiple sites across the genome acting in concert with environmental factors (4–6). The heritability of T2DM has been estimated to be 40–70% (7, 8), and more than 500 distinct genetic loci have been implicated with T2DM risk (6, 9–12). As T2DM is greatly impacted by genetics, genomic information has the potential to not only aid with early disease diagnosis but importantly also to stratify patients across disease subtypes (13) to initiate treatment intervention and lifestyle changes early in the disease progression.

During the last decade, an enormous effort has been in method development and construction of disease risk scores based on genomic information (14–17). However, until recently, these genome-wide genetic risk scores (GRS) have mainly been constructed using a single-trait approach. Because much of the variation within the human genome contributes to a large number of different complex traits and diseases (18), the accuracy of risk stratification can be improved by developing multi-trait (MT)-GRS accounting for the genetic correlation *among* traits. Using correlated information to construct GRS has theoretically—and to a minor extend empirical—been shown to increase the accuracy of disease risk prediction (6–8). T2DM is strongly correlated with a range of complex diseases and traits, such as overweight (19), cardiovascular disease (1, 19–21), hypertension (19, 22), and chronic kidney disease (19, 23); hence, T2DM is an excellent case for developing accurate GRS by leveraging correlated information.

The objective of the current study was to investigate the predictive performance of a MT-GRS model that combines marker effects from genome-wide association studies (GWAS) of T2DM and a number of correlated traits. The types of information included in this study were body mass index (BMI), height, smoking status, pulse rate, diastolic and systolic blood pressure, and a quantity of current medication use, as the total count of different prescription and over-the-counter medications is a proxy for general health and disease status. The aim of the present study was to investigate whether a MT-GRS model based on loci for multiple correlated traits had increased predictive discriminative power compared with a traditional single-trait (ST)-GRS model. This strategy was first applied within the UK Biobank (UKB) (24), and then extended to include information on two UKB-independent GWAS summary statistics and, finally, a combined model incorporating information from the UKB and the two independent T2DM GWAS data sets.

## Materials and Methods

### Phenotype and Genotype Data

Only unrelated British Caucasian individuals from the UKB (24) (*n* = 335,652 subjects) were used in the current study (excluding individuals with more than 5,000 missing genotype values or if having chromosomal aneuploidy). T2DM status was determined based on in-hospital records (by ICD-10 E.11, UKB data field 41270, which contains both main and secondary diagnoses) and self-reported disease state (UKB data field 20002) counting a total of 18,809 individuals. Seven additional phenotypes were also included: standing height, BMI, diastolic and systolic blood pressure, pulse rate, smoking status, and current medication use (measured as the number of different prescription and over-the-counter medications taken). These phenotypes were all adjusted for sex, age, UKB assessment center, and the first 10 genetic principal components (to account for any cryptic relatedness that were not accounted for by restricting to unrelated Caucasian British individuals), following inverse rank normalization to approximate normality.

Genotyped variants with minor allele frequency <0.01, genotype missingness >5%, or variants within the major histocompatibility complex were excluded from the analyses, resulting in a total of 599,297 genetic variants.

### Prediction of Diabetes Risk

**Figure 1:**
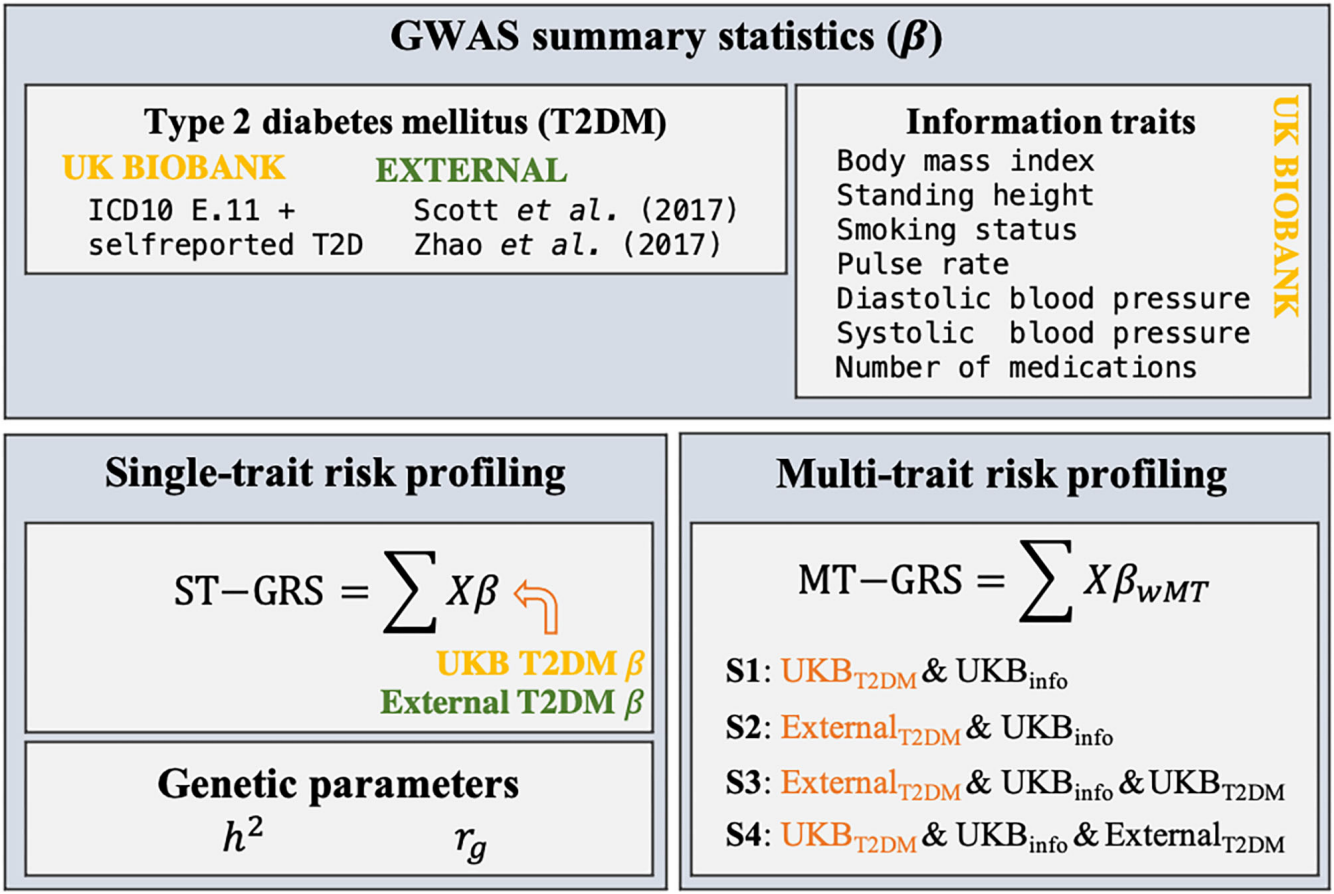
Schematic overview of the research design of the current study. Summary statistics (β) for T2DM and seven information traits were estimated from individual-level genotypic information (*X*) within the UKB using a 10-fold cross validation scheme. Two external GWAS summary statistics were identified. ST-GRS for T2DM was computed based on either the summary statistics obtained within the UKB or from the two external data sets. Estimates of the heritability (*h*^2^) and genetic correlations (*r*_*g*_) were estimated for T2DM, the seven information traits, and the two external T2DM studies. MT-GRS were computed based on four scenarios (S1–S4), depending on which types of information the predictor variable was adjusted for.

T2DM risk was determined using GRS based on either summary statistics obtained within the UKB cohort and other T2DM-related GWAS studies (Table 1). The overall workflow is depicted in Figure 1 and is described in detail below.

**Table 1:** Type 2 diabetes studies with available GWAS summary statistics independent of UKB.

**Study**	**References**	**n_**total**_**	**n_**case**_**	**m_**total**_**	**m_**UKB**_**
Scott et al. (2017)	(10)	159,208	26,676	12,056,346	595,528
Zhao et al. (2017)	(25)	265,678	73,337	8,796,184	558,105

*n_total_ is the sample size of the listed study. n_case_ is the number of T2DM cases within the listed study. m_total_ is the number of genetic variants used in the GWAS of the listed study. m_UKB_ is the number of variants in the listed study that were among the 599,297 quality-controlled genotyped variants in the UKB*.

#### UKB Summary Statistics

The White-British UKB cohort of unrelated individuals (335,652 subjects) was split into 10 folds with no overlap of samples within each fold, and for each fold, the marker effects for T2DM, standing height, BMI, diastolic and systolic blood pressure, pulse rate, smoking status, and current medication use, were estimated using logistic or linear regression as implemented in PLINK2 (26). In all analyses, the same set of covariates were included as those used during phenotypic adjustment as this has been shown to increase statistical power (27).

#### Publicly Available Type 2 Diabetes Summary Statistics

Two recently published GWAS for T2DM were identified (Table 1). Common for the studies were that they did not include UKB data, and therefore provide an independent training set. The regression coefficients were flipped such that the marker effect of the effect allele matched the effect allele within the UKB data.

#### Estimation of Genetic Parameters

Linkage disequilibrium (LD) between the genotyped variants was estimated as the squared Pearson's correlation coefficient (*r*^2^) between two genetic variants adjusted for sample size (*N*) as the standard estimator of the Pearson's correlation coefficient has an upward bias (28). The adjusted squared Pearson's correlation coefficient (${\tilde{r}}^{2}$) is obtained as (28):

(1) $${\tilde{r}}^{2}=r^{2}-\frac{1-r^{2}}{N-2},$$

which was computed with the R package qgg (29). LD scores (*l*) for all variants within a window size of 5,000 markers (2,500 markers around the *i*-th variant) were computed as

(2) $$l_{i}=\sum_{k=1}^{m=5000}{\tilde{r}}_{i,k}^{2}.$$

The MT-GRS model relies on selection index theory to obtain marker weights that require estimates of genetic parameters (30). The heritability (*h*^2^) and the genetic correlation (*r*_*g*_) between traits can be computed based on GWAS summary statistics using LD score regression (28). The heritability was estimated as the regression of the summary statistics on the LD score:

(3) $${\hat{h}}^{2}={\left(Z^{\prime }Z\right)}^{-1}\left(Z^{\prime }y\right),$$

where ***Z*** = n_*eff*_ × *l*/*m*, with *l* being the LD score (see Equation 2), *m* is the number of genetic variants, and *n*_*eff*_ is the effective number of individuals and is ${\text{n}}_{eff}=median\left(1/2\times af\times (1-af)\times SE{\left(\hat{b}\right)}^{2}\right)$, where *af* is the allele frequency, and $SE\left(\hat{b}\right)$ is the estimated standard error of the marker regression estimate. The response variable is $y={\left(\frac{\hat{b}}{SE\left(\hat{b}\right)}\right)}^{2}$, where $\hat{b}$ is the estimated regression coefficient for the genetic variants [for binary traits, the odds ratios (ORs) were converted to $\hat{b}=\log\left(OR\right)$, and $SE\left(\hat{b}\right)=\left|\;\hat{b}/P\left(X<\left(1-p\right)/2\right)\right|$, where *P*(*X* < (1 − *p*)/2) is the normal cumulative distribution given the marker *P*-value, *p* (31)]. Similarly, the genetic correlation between traits 1 and 2 can be estimated as:

(4) $${\hat{r}}_{g}=\frac{{\left(Z^{\prime }Z\right)}^{-1}\left(Z^{\prime }y\right)}{\sqrt{{\hat{h}}_{1}^{2}}\sqrt{{\hat{h}}_{2}^{2}}},$$

where $Z=\sqrt{n_{1}}\sqrt{n_{2}}\times \frac{l}{m}$, and $y=\frac{{\hat{b}}_{1}}{SE\left({\hat{b}}_{1}\right)}\times \frac{{\hat{b}}_{2}}{SE\left({\hat{b}}_{2}\right)}$. LD score regression was implemented in the R package qgg (29) and was computed for each of the 10-folds of random data subdivisions for T2DM and the seven information traits (Table 2), and among the information traits and the publicly available T2DM summary statistics (Table 1).

**Table 2:** UKB cohort description (*n* = 335,652) of T2DM cases and controls (count (%) or mean ± standard deviation).

**Characteristics**	**Controls**	**T2DM cases**	**Information trait**
*N*	316,935	18,809	
Age (years)	56.4 ± 8.0	60.5 ± 6.7	
Sex, male	144,070 (45.5%)	11,693 (62.2%)	
BMI (kg/m^2^)	27.1 ± 4.5	31.9 ± 5.8	X
Height (cm)	168.8 ± 9.2	170.0 ± 9.3	X
Pulse rate (BPM)	69.1 ± 11.1	73.6 ± 13.1	X
Systolic blood pressure (mmHg)	138.0 ± 18.6	142.6 ± 18.0	X
Diastolic blood pressure (mmHg)	82.3 ± 10.1	82.3 ± 10.3	X
Smoking status			X
Never	175,002 (55.4%)	7,687 (41.1%)	
Former	109,007 (34.5%)	8,663 (46.3%)	
Current	31,867 (10.1%)	2,345 (12.6%)	
Number of medications	2.3 ± 2.4	5.7 ± 3.7	X

#### ST-GRS

The ST-GRS was computed as,

(5) $$\text{ST}-\text{GRS}=\sum_{i=1}^{m}X_{i}{\hat{b}}_{i},$$

where ***X***_*i*_ is the *i*-th column of the genotype matrix containing allelic counts, ${\hat{b}}_{i}$ is the estimated marker effect for the *i*-th marker, and *m* is the number of variants left after LD pruning (*r*^2^ < 0.1, <0.5, or <0.9) and *P*-value thresholding (*P* < 0.001, 0.01, 0.05, 0.1, 0.2, 0.3, 0.5, 0.7, 0.9, and 0.99). The genetic scoring was performed with the R package qgg (29).

#### MT-GRS

The accuracy of GRS can be improved by leveraging information from correlated traits by adjusting the marker effects ($\hat{b}$) (30). The adjustment of the marker effects for the focal trait (*f*, i.e., T2DM) is obtained by computing index weights for each marker ($w_{i}\prime$)

(6) $${\hat{b}}_{wMT_{i}}=w_{i}\prime {\hat{b}}_{i}.$$

From quantitative genetic theory, selection indices have been developed for MT selection, in which many ST individual genetic effects (i.e., breeding values) are combined with an index weight allowing selection of the individuals with the best MT phenotype (32, 33). The optimal weights can be derived as ***w*** = ***V***^−1^***C***, where ***C*** is a *k* × 1 column vector of covariances between the $\hat{b}$ values of the *k* traits and the true marker effects of the focal trait (***b***_*f*_), and ***V*** is a *k* × *k* variance–covariance **matrix** of the $\hat{b}$ values:

(7) $$w={\left[\begin{array}{ccc} var({\hat{b}}_{1}) & \;\ldots & \;cov({\hat{b}}_{1},{\hat{b}}_{k})\\ \vdots & \;\ddots & \;\vdots \\ cov({\hat{b}}_{k},{\hat{b}}_{1}) & \;\ldots & \;var({\hat{b}}_{k}) \end{array}\right]}^{-1}\;\left[\begin{array}{c} cov(b_{f},{\hat{b}}_{1})\\ \ldots \\ cov(b_{f},{\hat{b}}_{k}) \end{array}\right].$$

The diagonal elements of variance–covariance matrix, ***V***, are

(8) $$var\left({\hat{b}}_{k}\right)=\frac{h_{k}^{2}}{M}+\frac{1}{N_{k}},$$

where *M* is the effective number of chromosomal segments [here *M* = 60, 000 (30, 34)] and *N*_*k*_ is the number of observations for trait *k*. The off-diagonal elements of ***V*** for trait *k* and *l* are

(9) $$cov\left({\hat{b}}_{k},{\hat{b}}_{l}\right)=\frac{r_{g}h_{k}h_{l}}{M},$$

which is the same for the elements of ***C***. Combining Equations (8) and (9), Equation (7) becomes

(10) $$w={\left[\begin{array}{ccc} \frac{h_{1}^{2}}{M}+\frac{1}{N_{1}} & \;\ldots & \;\frac{r_{g}h_{1}h_{k}}{M}\\ \vdots & \;\ddots & \;\vdots \\ \frac{r_{g}h_{k}h_{1}}{M} & \;\ldots & \;\frac{h_{k}^{2}}{M}+\frac{1}{N_{k}} \end{array}\right]}^{-1}\;\left[\begin{array}{c} \frac{h_{1}^{2}}{M}\\ \ldots \\ \frac{r_{g}h_{1}h_{k}}{M} \end{array}\right].$$

The MT-GRS is then obtained as the sum of adjusted marker effects,

(11) $$\text{MT}-\text{GRS}=\sum_{i=1}^{m}X_{i}{\hat{b}}_{wMT_{i}}.$$

MT-GRS was computed by applying LD pruning (*r*^2^ < 0.1, <0.5, or <0.9) and *P*-value thresholding (*P* < 0.001, 0.01, 0.05, 0.1, 0.2, 0.5, 0.75, and 0.99) based on UKB genotypes and T2DM summary statistics; thus, the same LD pruning and *P*-value thresholding were applied across traits.

Four MT scenarios were applied, resulting in four different predictors (Figure 1): (1) UKB T2DM summary statistics combined with the seven UKB information traits; (2) external T2DM summary statistics [i.e., results from Scott et al. (10) and Zhao et al. (25)] combined with the seven UKB information traits; (3) external T2DM summary statistics combined with the seven UKB information traits and UKB T2DM summary statistics; and (4) UKB T2DM summary statistics combined with the seven UKB information traits and the two external T2DM summary statistics.

#### GRS Accuracy

The accuracy of ST-GRS and MT-GRS was determined using Nagelkerke's variance explained (*R*^2^),

(12) $$R^{2}=\frac{1-e^{-LR/n}}{1-e^{-(-2L_{0})/n}}$$

where LR is the likelihood ratio comparing two nested logistic regression models, *L*_0_ is the log-likelihood of a model neglecting the GRS, and *n* is the number of observations. The full model included sex, age, UKB assessment center, the first 10 genetic principal components, and the GRS, whereas the reduced model did not contain the GRS effect. For visualization, the GRS were divided into percentiles, and the disease prevalence within each bin was computed; the OR for each percentile was computed adjusting for sex, age, UKB assessment center, and the first 10 genetic principal components, and the OR was expressed relative to the 50-th percentile.

## Results

### ST Prediction and Genetic Parameters

The analysis of T2DM was performed using 335,662 unrelated individuals from UKB with more than 18,000 T2DM cases (Table 2). A larger proportion of T2DM cases were males and smokers; on average, T2DM cases were older than individuals without T2DM, had higher BMI, and on average used more medications than non-diabetic individuals (Table 2).

**Figure 2:**
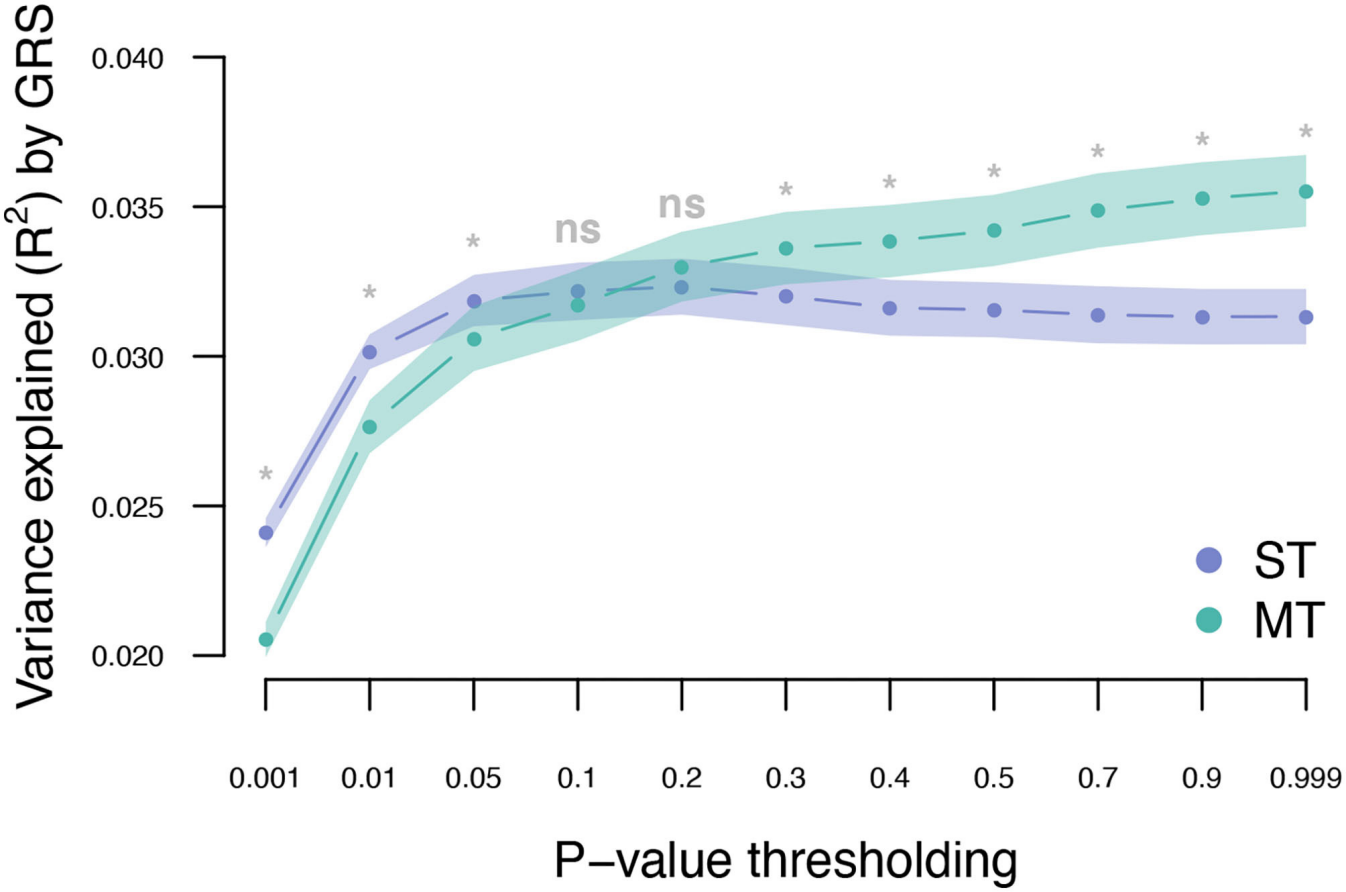
Variance explained (*R*^2^) for type 2 diabetes by ST-GRS and MT-GRS (LD pruning *r*^2^ < 0.9) using *P*-value thresholding (X-axis). Points indicate mean *R*^2^ for a given threshold, and the surrounding shading indicates the standard error of the mean. ns, non-significant difference between ST and MT, *significant difference between ST and MT.

The UKB cohort was split into 10 training and validation sets, and within-cohort marginal marker effects of common genotyped variants were estimated for each training set. After LD pruning and *P*-value thresholding, ST-GRS were computed for individuals within the validation sets. The maximum prediction accuracy for ST-GRS was *R*^2^ = 0.032 when using variants with LD *r*^2^ < 0.9 and *P* < 0.05 (Figure 2; Supplementary Table 2).

**Figure 3:**
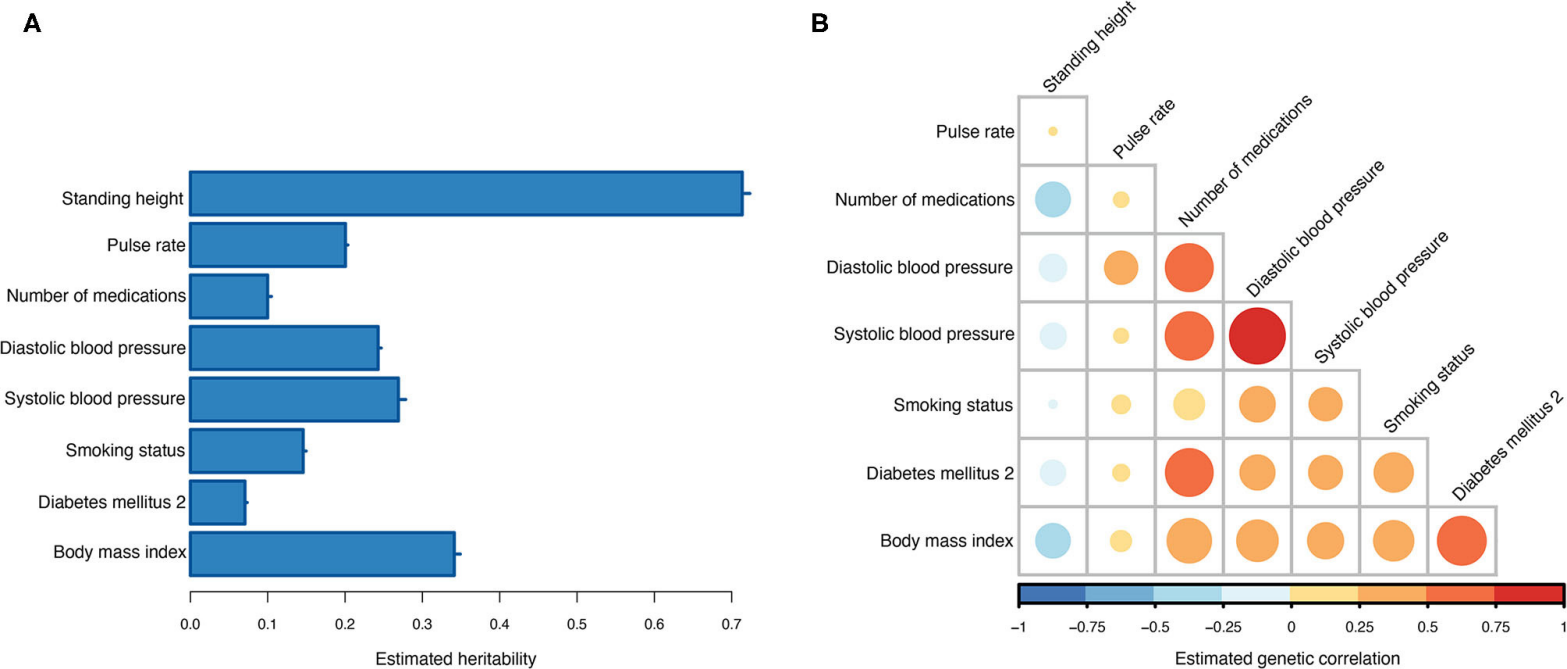
Estimated genetic parameters. **(A)** Estimated heritabilities for T2DM and the seven information traits. Errors bars indicate the standard error of the estimates across the 10 training sets. **(B)** Estimated genetic correlations between T2DM and the seven information traits.

Across the 10 training sets, the average heritability for T2DM on the observed scale was 0.07 (0.31 on the liability scale). Seven information traits were included and used in the MT genetic risk scoring (Table 2). All seven traits showed non-zero heritability estimates (Figure 3A), and the strongest genetic correlation was observed between diastolic and systolic blood pressure (Figure 3B). Current medication use was the trait that showed the highest genetic correlation to most of the other traits, and only standing height showed negative genetic correlation to the other traits (Figure 3B).

### Leveraging Correlated Information for MT Prediction

The T2DM marginal effects were adjusted using the estimated genetic parameters to compute MT-GRS (Scenario 1; Figure 1). Across the three levels of LD pruning, the predictive ability was generally improved when the marginal SNP effects were adjusted by the seven information traits (Supplementary Figure 1; Supplementary Table 2). The highest prediction accuracy (*R*^2^ = 0.036) was obtained at LD *r*^2^ < 0.9 and *P* < 0.999 (Figure 2; Supplementary Table 2), which corresponds to an improved prediction accuracy by 12.5%

**Figure 4:**
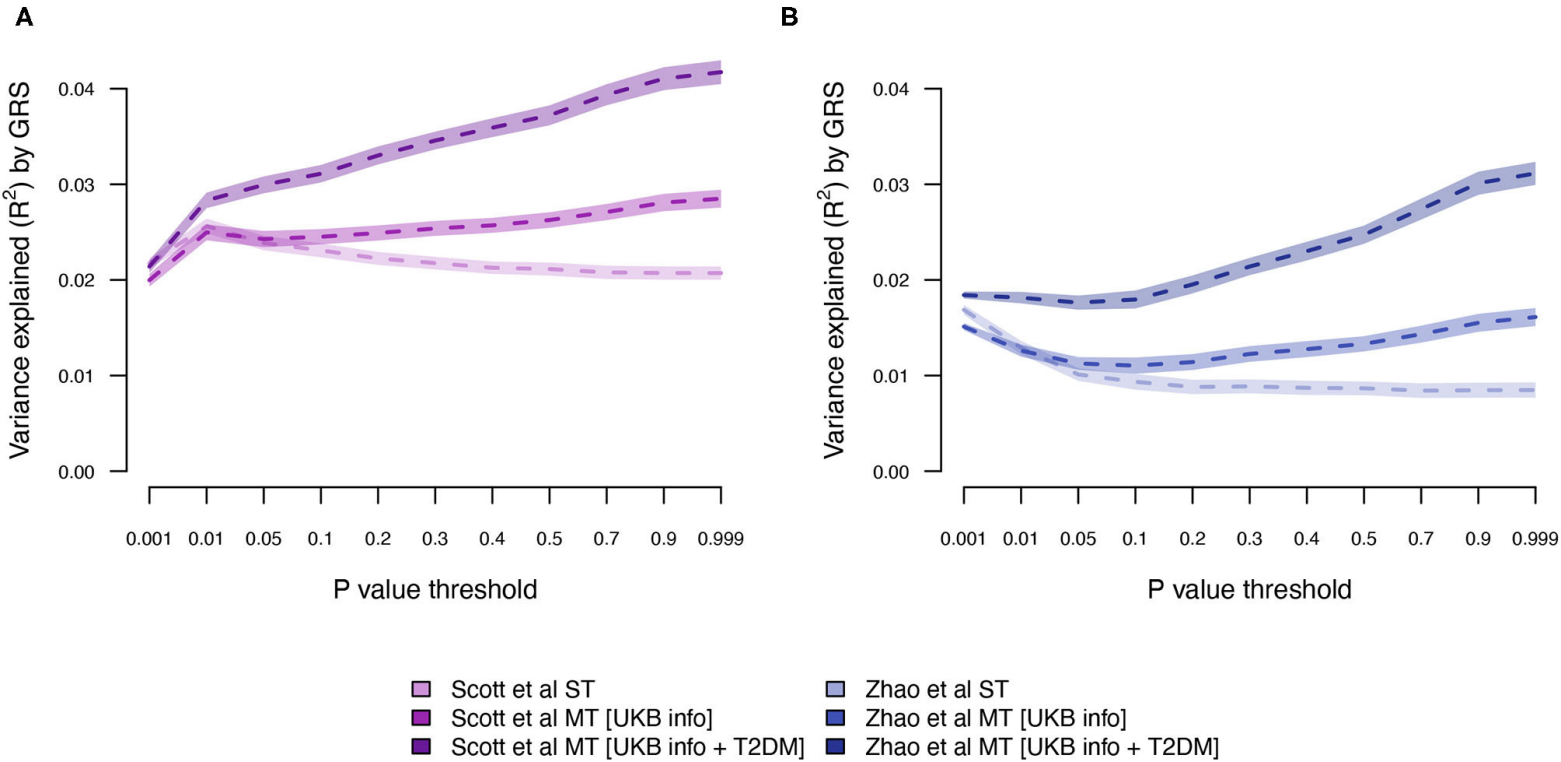
Variance explained (*R*^2^) for type 2 diabetes by ST-GRS and MT-GRS (LD pruning *r*^2^ < 0.9) using publicly available summary statistics from **(A)** Scott et al. (10) and **(B)** Zhao et al. (25). Statistics of model comparisons are found in Supplementary Tables 3, 4.

Next, we estimated the T2DM risk within the UKB using summary statistics from two independent external sets of summary statistics (Figure 1). Both external data sets [Scott et al. (10) and Zhao et al. (25)] showed low prediction accuracy when the GRS solely were computed using T2DM summary statistics [Scott et al. (10): *R*^2^ = 0.026 at LD *r*^2^ = 0.9 and *P* < 0.01; and Zhao et al. (25): *R*^2^ = 0.017 at LD *r*^2^ = 0.9 and *P* < 0.001; Figure 4; Supplementary Tables 3, 4; Supplementary Figure 2]. The external T2DM summary statistics were adjusted using summary statistics from the seven information traits obtained from the UKB (Scenario 2; Figure 1; Supplementary Table 1; Supplementary Figure 3), which for the summary statistics from Scott et al. (10) increased the prediction accuracy by 8%, but for Zhao et al. (25), a marginal drop in accuracy was observed when comparing the local maximum for ST-GRS with the local maximum for MT-GRS [*R*^2^ = 0.017 (*r*^2^ = 0.9, *P* < 0.001)] vs. 0.016 [*R*^2^ = 0.016 (*r*^2^ = 0.9, *P* < 0.999); Supplementary Table 4]; however, comparing the accuracy within the *P*-value threshold, the accuracy of the MT-GRS model was superior over the ST (Supplementary Table 4). Extending the MT model to also include UKB T2DM summary statistics (Scenario 3, Figure 1), the accuracy was further increased by 50% (from 0.028 to 0.042; Figure 4) and 94% (from 0.016 to 0.031; Figure 4) using the summary statistics of Scott et al. (10) and Zhao et al. (25), respectively.

**Figure 5:**
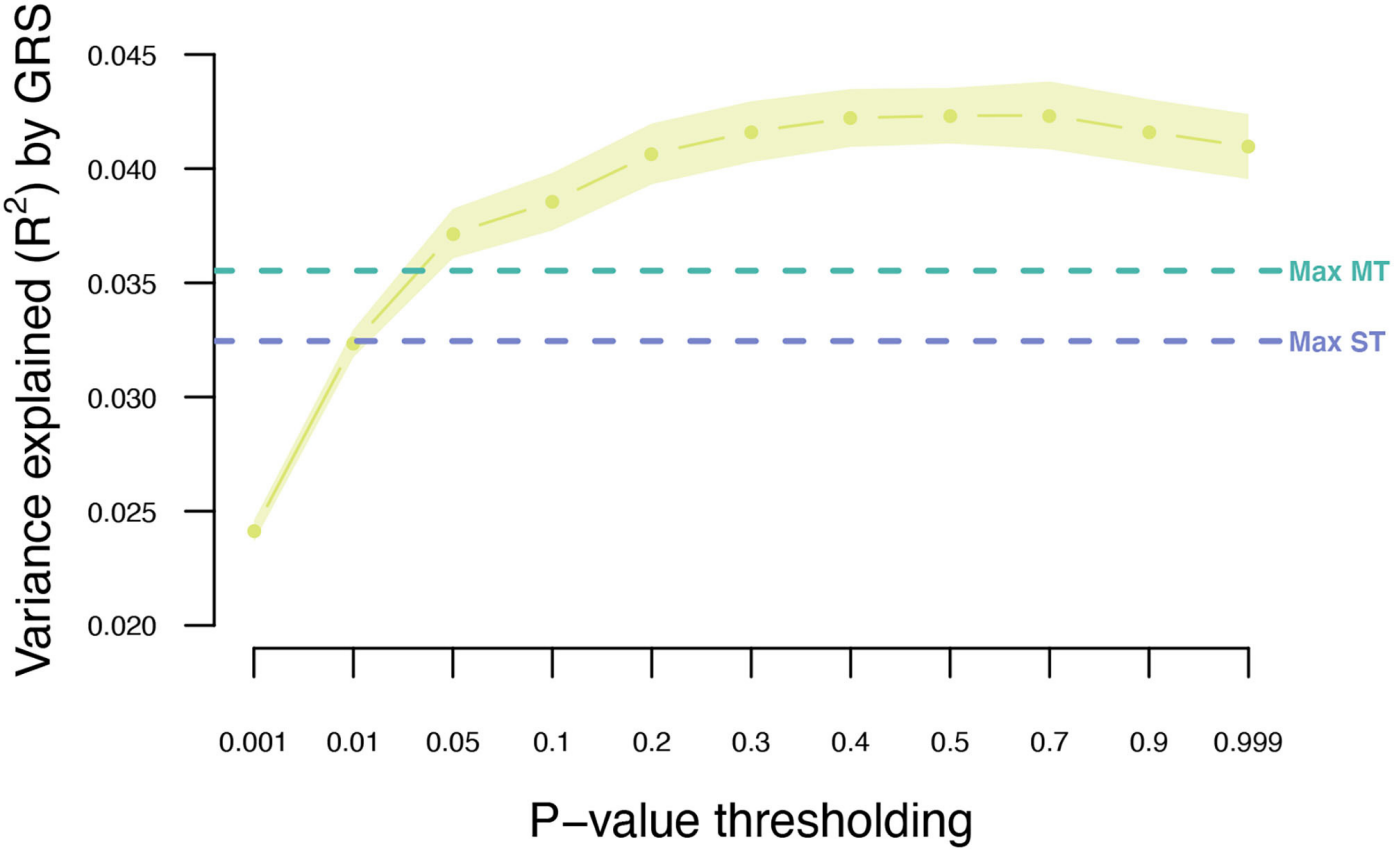
Variance explained (*R*^2^) for type 2 diabetes using MT model with the seven information traits and publicly available T2DM summary statistics. Points indicate mean *R*^2^ for a given threshold, and the surrounding shading indicates the standard error of the mean. The horizontal dashed lines indicate the maximum *R*^2^ obtained for ST-GRS and MT-GRS without publicly available summary statistics.

The MT model trained within the UKB was further extended to also include summary statistics from the two independent T2DM GWAS data sets (Scenario 4; Figure 1). Adjusting the UKB T2DM summary statistics by the seven information traits and the two independent T2DM GWAS data sets resulted in an increase in prediction accuracy from 0.032 to 0.043 (Figure 5; Supplementary Table 2), which is an increase of 34%.

### T2DM Risk Stratification

**Figure 6:**
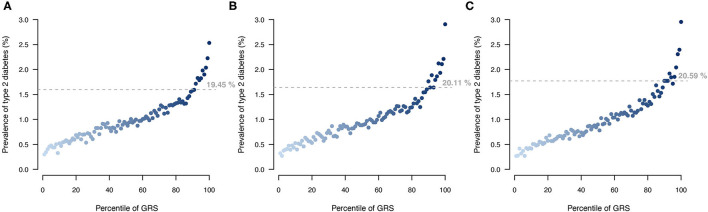
Comparison of T2DM risk gradient within the UKB according to GRS percentile for **(A)** ST model, **(B)** MT model using the seven information traits, and **(C)** MT model with the seven information traits and the T2DM testing data. Each point indicates the average T2DM prevalence within each percentile of GRS across then 10 validation sets. Horizontal lines indicate the prevalence at the top 10 GRS percentile, and percentage indicates the prevalence among the top 10% with the highest genetic risk.

Stratifying UKB participants based on their T2DM genetic risk showed that a larger proportion of individuals with a T2DM diagnosis were among the top 10% of individuals with highest genetic score when applying the MT strategy (Figure 6). The MT-GRS that in addition to the seven information traits also included information from the independent testing data gave a better stratification of cases by distributing a larger proportion of T2DM cases within the top risk (Figure 6), which also was apparent with a large OR of the top 10% compared to the remaining (Supplementary Figure 4).

## Discussion

Precision medicine is predicted to change the way we prevent, diagnose, risk stratify individuals, and treat medical conditions (35, 36) through development of targeted preventive or treatment approaches based on the genetic background, biomarkers, environmental exposures, and lifestyle of the individual. Diagnosis and treatment plans based on genetic testing has been effectively applied to several monogenic disorders (37); however, for common complex diseases, genomic information has been far less incorporated. One reason for the lack of incorporating genomic information in disease prevention and diagnosis for complex diseases is because a large proportion of the underlying genetic variation remains unexplained (38, 39). In the current study, we investigated whether an MT-GRS approach provided more accurate risk stratification than traditional ST genetic scoring approaches.

Adjusting the UKB T2DM marker effects by the genomic correlation of the seven information traits increased the prediction accuracy from *R*^2^ = 0.032 to 0.036, and further adjusted by the two UKB-independent T2DM studies increased the accuracy to *R*^2^ = 0.042. The great improvement in prediction accuracy (31%) is achieved as a consequence of abundant genomic pleiotropy (18, 30) and the apparent genomic correlation with the selected traits. In comparison, Khera et al. (14) reported a prediction accuracy of ST-GRS of *R*^2^ = 0.028 (14), and Maier et al. (30) obtained an accuracy of *R*^2^ < 0.01 for both ST-GRS and MT-GRS (30). Although Maier et al. (30) showed increased prediction accuracy by combining the marker effects of selected traits (30), our reported prediction accuracies were greatly elevated compared with Maier et al. (30), most likely driven by differences in the included traits, and thereby in the optimal weights caused by differences in genomic correlation among the traits.

One of the information traits we included in the MT-GRS was the genetic liability to current medication use, which is the number of different medications the UKB participants have taken at the time of the verbal interview. Because most individuals that suffers from temporary or chronic diseases will undergo medical intervention and because of comorbidity many individuals will have multiple medical conditions, those individuals will be treated with a range of different medicines. Consequently, the total set of prescription and over-the-counter drugs is potentially an informative index of the current medical and health status of an individual. Wu et al. (40) performed genetic analysis of self-reported medication use within the UKB and found that categories of different types of medication were strongly genetically associated with a range of different diseases and traits (40). We found that the genetic correlation between T2DM and medication use was r_g_ = 0.55 (only the correlation between T2DM and BMI had higher estimate, r_g_ = 0.58). This is also evident by investigating the optimal weights (Equation 7), where BMI and medication use were the two information traits with the largest weights (Supplementary Figure 5A), besides T2DM itself. Including summary statistics from the two published T2DM association studies only marginally affected the optimal weights (Supplementary Figure 5B).

Although the exact level of prediction accuracy of T2DM was considerably lower when using external data from Zhao et al. (25) compared to data from Scott et al. (10) (Figure 4), the percentage increase when extending ST-GRS to the MT-GRS was higher for Zhao et al. (25) (82%) compared with Scott et al. (10) (62%), despite the much greater sample size by Zhao et al. (25) (Table 1). The discrepancy in prediction accuracy is most likely a consequence of different ancestries of the two external T2DM studies (10, 25), where the ancestry of the individuals in the study by Scott et al. (10) is more similar to the ancestry of the UKB (European) than the study by Zhao et al. (25) (mixed ancestry). It is well-established that across ancestry, risk prediction is very difficult because the LD between populations is very diverse (41–43).

The last decade has shown us that the sample size of human genetic association studies keeps increasing (44, 45), not only entailing more association signals but also providing more accurate effect estimates. This in conjunction with the increasingly accessibility of publicly available GWAS summary statistics (46, 47) implies that genomic prediction of complex diseases will continually improve, in particular if multivariate predictors are created by integrating information across studies. Although we have demonstrated increased prediction accuracy by constructing MT-GRS, our work has several limitations. Firstly, as our training data were the UKB and with a 10-fold cross-validation scheme, the number of cases became limited, meaning less accurate marker effect estimation and thereby less accurate risk stratification. Secondly, although we in addition to the UKB summary statistics from the 10-fold cross-validation obtained T2DM summary statistics from two independent studies (Table 1), we only had access to genotype information from the UKB and no other T2DM cohorts. Thirdly, we restricted the number of information traits to seven (Table 2), based on the criterion that it should be a type of information that is easy and accurate to measure and obtain; height, BMI, pulse rate, and diastolic and systolic blood pressure are things that we easily and accurately can measure, and smoking status and current medication use can easily be obtained by asking the participants. Accurate observations lead to more accurate estimation of marker effects and thereby better prediction accuracies. It is compelling to speculate whether other types of information traits would improve prediction accuracy even more, and additional studies are warranted for developing methods for identifying the set of information traits most important for a particular disease.

Genomic information has the potential to change the way we diagnose and treat individuals today and will be central for implementing preventive healthcare in the clinics. An important aspect of precision medicine is accurate prediction of genetic risk toward common diseases, as it may guide the general practitioners to better and earlier identify those individuals who have an inherent genetically lifetime high disease risk, and then to initiate lifestyle changes potentially before disease outcome. Moreover, precise stratification of T2DM patients not only based on their pathophysiological symptoms (13) but also on their genetic makeup may help the general practitioners to treat high-risk patients more aggressively, which has the potential to slow down disease progression, reduce symptoms, and prevent severe morbidity and mortality.

In conclusion, by incorporating information traits and two previously published T2DM GWAS results, the prediction accuracy for T2DM was increased by 31% (from *R*^2^ = 0.032 to *R*^2^ = 0.042), clearly demonstrating the added benefit of incorporating correlated information in the construction of GRS. Thus, incorporating genomic information on correlated traits and disease is advisable for obtaining improved individual genetic risk stratification.

## Data Availability Statement

Publicly available datasets were analyzed in this study. This data can be found at: The genetic and phenotypic data were obtained from the UK Biobank Resource (ID 31269). Researchers can apply for access through: https://www.ukbiobank.ac.uk/registerapply/. Summary statistics for T2DM were obtained from published studies.

## Ethics Statement

The studies involving human participants were reviewed and approved by The Ethics and Governance Framework (EGF) sets standards for the UK Biobank project so that all necessary safeguards are in place to ensure that the data and samples are only used for scientifically and ethically approved research. Participants of the UK Biobank have given their consent to participate which will apply throughout the lifetime of the UK Biobank unless the participants withdraw. Their consent involves the collection and storage of biological material (blood, saliva, urine samples) as well as collection of electronic health records (GP, hospitals, dental and prescription records). Information on the individual data level is anonymised for the researchers, and every research project has its own anonymised data. The ethics committee waived the requirement of written informed consent for participation.

## Author Contributions

PDR and PS conceived and designed the research project and performed the genetic analyses. PDR, PS, MN, and MK interpreted the results. All authors contributed to the preparation of the manuscript, read, edited, and approved the manuscript.

## Funding

PDR has received funding from The Lundbeck Foundation (R287-2018-735).

## Conflict of Interest

The authors declare that the research was conducted in the absence of any commercial or financial relationships that could be construed as a potential conflict of interest.

## Publisher's Note

All claims expressed in this article are solely those of the authors and do not necessarily represent those of their affiliated organizations, or those of the publisher, the editors and the reviewers. Any product that may be evaluated in this article, or claim that may be made by its manufacturer, is not guaranteed or endorsed by the publisher.
