# Supplementary material for: Multi-Trait Genomic Risk Stratification for Type 2 Diabetes
Source: Front Med (Lausanne). 2021 Sep 8;8:711208. doi: 10.3389/fmed.2021.711208 (PMC8455930; doi:10.3389/fmed.2021.711208)
Supplement: Supplementary file 1 [file Data_Sheet_1.PDF]

Supplementary material for

Multi-Trait Genomic Risk Stratification for Type 2 Diabetes

Palle Duun Rohde[1,2,*], Mette Nyegaard[2,3], Mads Kjolby[3,4,5,6] & Peter Sørensen[7]

1 Department of Chemistry and Bioscience, Aalborg University, Aalborg, Denmark

2 Department of Health Science and Technology, Aalborg University, Aalborg, Denmark

3 Department of Biomedicine, Aarhus University, Aarhus, Denmark

4 Department of Population Health and Genomics, University of Dundee, Dundee, Scotland

5 Department of Clinical Pharmacology, Aarhus University Hospital, Aarhus, Denmark

6 Steno Diabetes Center Aarhus, Aarhus University Hospital, Aarhus, Denmark

7 Centre for Quantitative Genetics and Genomics, Aarhus University, Aarhus, Denmark

* Correspondence: Palle Duun Rohde, palledr@bio.aau.dk

The supplementary material contains the following:

Supplementary Tables

Table S1	Heritability estimates for type 2 diabetes mellitus (T2DM).
Table S2	Summary of average variance explained for T2DM in the UKB.
Table S3	Summary of average variance explained for T2DM in the UKB using Scott et al (2017) summary statistics.
Table S4	Summary of average variance explained for T2DM in the UKB using Zhao et al (2017) summary statistics.

Supplementary Figures

Figure S1	Average variance explained for T2DM in the UKB.
Figure S2	Average variance explained for T2DM in the UKB using publicly available summary statistics
Figure S3	Estimated genetic correlations between UKB info traits and T2DM summary statistics from literature.
Figure S4	Risk gradient for T2DM.
Figure S5	Selection weights across the ten training sets.

Table S1 | Heritability estimates for type 2 diabetes on the observed and on the liability scale.

Study	Estimated heritability	
	**Observed**	**Liability***
Rohde et al 2021	0.07	0.31
Scott et al 2017	0.19	0.32
Zhao et al 2017	0.02	0.02

* Assuming disease prevalence in the UK of 7%

Table S2 | Summary of average variance explained (R^2) for T2DM in the UKB for the single-trait (ST) and multi-trait (MT) genetic risk scores (GRS), and comparison of R^2 for ST and MT models by *t*-test.

LD pruning r^2	P threshold	R^2 ST	R^2 MT	R^2 MT2	P-val*	P-val†	P-val‡
10	0.001	0.024	0.021	0.024	1.78E-05	8.06E-01	2.45E-05
10	0.01	0.029	0.027	0.032	3.73E-04	3.24E-06	1.80E-07
10	0.05	0.031	0.030	0.037	6.18E-02	1.01E-05	1.25E-08
10	0.1	0.031	0.031	0.038	8.99E-01	1.49E-05	2.26E-07
10	0.2	0.031	0.032	0.040	4.48E-02	1.10E-05	1.81E-06
10	0.3	0.031	0.033	0.041	2.91E-03	9.58E-06	3.22E-06
10	0.4	0.031	0.033	0.042	8.35E-04	3.28E-06	1.12E-06
10	0.5	0.031	0.033	0.042	5.17E-04	2.44E-06	1.05E-06
10	0.7	0.031	0.034	0.043	1.78E-04	5.53E-06	7.70E-06
10	0.9	0.031	0.034	0.043	1.95E-04	2.58E-06	3.93E-06
10	0.999	0.031	0.034	0.043	1.22E-04	2.69E-06	3.66E-06
50	0.001	0.024	0.020	0.024	5.43E-06	6.24E-01	8.62E-07
50	0.01	0.030	0.028	0.032	9.22E-05	1.10E-05	3.12E-07
50	0.05	0.032	0.031	0.037	2.01E-02	1.00E-05	1.99E-10
50	0.1	0.032	0.032	0.038	3.33E-01	2.39E-05	1.09E-07
50	0.2	0.032	0.033	0.040	1.49E-01	8.27E-06	7.19E-07
50	0.3	0.032	0.034	0.041	6.06E-03	2.93E-06	6.27E-07
50	0.4	0.031	0.034	0.042	1.01E-03	5.02E-07	2.60E-07
50	0.5	0.031	0.034	0.043	4.25E-04	4.76E-07	5.11E-07
50	0.7	0.031	0.035	0.043	9.08E-05	1.66E-06	7.39E-06
50	0.9	0.031	0.035	0.042	5.81E-05	2.27E-06	2.88E-05
50	0.999	0.031	0.035	0.042	3.57E-05	2.26E-06	5.15E-05
90	0.001	0.024	0.021	0.024	7.12E-06	9.06E-01	1.56E-06
90	0.01	0.030	0.028	0.032	1.23E-04	3.06E-05	5.34E-07
90	0.05	0.032	0.031	0.037	1.61E-02	3.38E-06	1.77E-10
90	0.1	0.032	0.032	0.039	3.03E-01	5.39E-06	2.98E-08
90	0.2	0.032	0.033	0.041	1.35E-01	2.27E-06	2.00E-07
90	0.3	0.032	0.034	0.042	3.95E-03	4.48E-07	1.14E-07
90	0.4	0.032	0.034	0.042	5.58E-04	2.14E-07	3.19E-07
90	0.5	0.032	0.034	0.042	2.08E-04	1.10E-07	3.75E-07
90	0.7	0.031	0.035	0.042	4.40E-05	1.60E-06	1.63E-05
90	0.9	0.031	0.035	0.042	3.12E-05	2.90E-06	1.17E-04
90	0.999	0.031	0.036	0.041	1.48E-05	3.61E-06	3.33E-04

ST: Single trait GRS

MT: Multi-trait GRS [UKB info traits]

MT2: Multi-trait GRS [UKB info traits + T2DM Scott+Zhao]

* P-value comparing ST and MT

† P-value comparing ST and MT2

‡ P-value comparing MT and MT2

Tabel 3| Summary of average variance explained (R^2) for T2DM in the UKBB based on summary statistics from Scott et al (2017) using single-trait (ST) and multi-trait (MT) genetic risk scores (GRS) (without and with T2DM summary statistics from UKB), and comparison of R^2 for ST and MT models by *t*-test.

LD Pruning r^2	P threshold	R^2 ST	R^2 MT	R^2 MT2	P-val*	P-val†	P-val‡
10	0.001	0.022	0.020	0.021	2.39E-05	1.42E-01	2.53E-08
10	0.01	0.026	0.025	0.028	3.57E-03	1.84E-06	7.45E-10
10	0.05	0.024	0.024	0.030	3.20E-01	8.39E-09	3.04E-11
10	0.1	0.023	0.024	0.030	2.50E-04	5.19E-09	1.75E-10
10	0.2	0.023	0.025	0.032	7.09E-06	1.90E-09	2.32E-10
10	0.3	0.023	0.026	0.034	1.07E-06	7.72E-10	1.08E-10
10	0.4	0.022	0.026	0.035	3.10E-07	6.62E-10	1.38E-10
10	0.5	0.022	0.026	0.036	6.84E-08	2.83E-10	7.47E-11
10	0.7	0.022	0.027	0.037	6.52E-08	2.67E-10	4.10E-11
10	0.9	0.022	0.027	0.038	6.54E-08	3.34E-10	3.72E-11
10	0.999	0.022	0.028	0.039	6.62E-08	4.66E-10	5.30E-11
50	0.001	0.022	0.020	0.021	2.09E-05	8.67E-02	6.68E-08
50	0.01	0.026	0.025	0.028	5.65E-03	7.55E-07	6.79E-10
50	0.05	0.024	0.024	0.030	8.83E-02	1.88E-09	1.61E-11
50	0.1	0.023	0.024	0.031	4.95E-05	1.44E-09	6.53E-11
50	0.2	0.022	0.025	0.033	2.31E-06	9.66E-10	7.62E-11
50	0.3	0.022	0.025	0.034	2.53E-07	2.81E-10	2.34E-11
50	0.4	0.021	0.026	0.036	9.97E-08	3.53E-10	6.49E-11
50	0.5	0.021	0.026	0.037	1.90E-08	1.93E-10	5.67E-11
50	0.7	0.021	0.027	0.039	1.79E-08	2.50E-10	6.18E-11
50	0.9	0.021	0.028	0.040	2.34E-08	4.86E-10	1.43E-10
50	0.999	0.021	0.028	0.041	2.26E-08	5.63E-10	1.98E-10
90	0.001	0.022	0.020	0.021	2.92E-05	1.17E-01	1.17E-07
90	0.01	0.026	0.025	0.028	1.02E-02	1.06E-06	7.64E-10
90	0.05	0.024	0.024	0.030	6.13E-02	1.98E-09	8.12E-12
90	0.1	0.023	0.025	0.031	3.76E-05	2.16E-09	1.13E-10
90	0.2	0.022	0.025	0.033	1.85E-06	8.37E-10	5.63E-11
90	0.3	0.022	0.025	0.035	1.78E-07	1.91E-10	9.85E-12
90	0.4	0.021	0.026	0.036	8.47E-08	2.92E-10	4.01E-11
90	0.5	0.021	0.026	0.037	1.33E-08	1.42E-10	4.02E-11
90	0.7	0.021	0.027	0.039	9.92E-09	1.96E-10	5.28E-11
90	0.9	0.021	0.028	0.041	1.61E-08	5.16E-10	1.72E-10
90	0.999	0.021	0.028	0.042	1.48E-08	4.98E-10	1.81E-10

ST: Single trait GRS

MT: Multi-trait GRS [UKB info traits]

MT2: Multi-trait GRS [UKB info traits + T2DM]

* P-value comparing ST and MT

† P-value comparing ST and MT2

‡ P-value comparing MT and MT2

Tabel 4| Summary of average variance explained (R^2) for T2DM in the UKBB based on summary statistics from Zhao et al (2017) using single-trait (ST) and multi-trait (MT) genetic risk scores (GRS) (without and with T2DM summary statistics from UKB), and comparison of R^2 for ST and MT models by *t*-test.

LD Pruning r^2	P threshold	R^2 ST	R^2 MT	R^2 MT2	P-val*	P-val†	P-val‡
10	0.001	0.017	0.015	0.018	2.53E-05	1.04E-03	3.23E-09
10	0.01	0.013	0.012	0.018	6.75E-02	6.74E-09	1.41E-12
10	0.05	0.010	0.011	0.017	3.35E-06	3.92E-12	4.18E-13
10	0.1	0.009	0.011	0.018	8.93E-08	4.08E-12	5.50E-12
10	0.2	0.009	0.011	0.019	5.34E-08	1.02E-11	2.74E-12
10	0.3	0.009	0.012	0.020	5.78E-09	3.07E-12	4.57E-12
10	0.4	0.009	0.012	0.021	6.76E-09	5.98E-12	2.83E-12
10	0.5	0.009	0.012	0.022	7.85E-09	1.15E-11	6.04E-12
10	0.7	0.009	0.013	0.024	1.49E-08	9.24E-11	2.96E-11
10	0.9	0.009	0.013	0.025	6.27E-09	1.03E-10	8.28E-11
10	0.999	0.009	0.014	0.026	5.38E-09	1.01E-10	8.36E-11
50	0.001	0.017	0.015	0.018	3.85E-05	1.27E-03	6.41E-09
50	0.01	0.013	0.013	0.018	1.16E-01	3.14E-09	7.62E-13
50	0.05	0.010	0.011	0.017	2.16E-06	3.40E-12	2.80E-13
50	0.1	0.009	0.011	0.018	6.36E-08	5.90E-12	1.08E-11
50	0.2	0.009	0.011	0.019	4.00E-08	1.29E-11	2.23E-12
50	0.3	0.009	0.012	0.021	1.52E-08	8.13E-12	2.65E-12
50	0.4	0.009	0.012	0.022	8.93E-09	2.82E-11	7.03E-12
50	0.5	0.009	0.013	0.024	1.26E-08	5.07E-11	1.05E-11
50	0.7	0.008	0.014	0.026	6.85E-09	8.41E-11	2.22E-11
50	0.9	0.008	0.015	0.029	4.67E-09	1.72E-10	1.03E-10
50	0.999	0.008	0.015	0.030	2.76E-09	1.31E-10	9.05E-11
90	0.001	0.017	0.015	0.018	3.98E-05	1.54E-03	1.10E-08
90	0.01	0.013	0.013	0.018	1.28E-01	2.99E-09	1.08E-12
90	0.05	0.010	0.011	0.018	2.59E-06	4.48E-12	3.98E-13
90	0.1	0.009	0.011	0.018	3.65E-08	3.73E-12	5.23E-12
90	0.2	0.009	0.011	0.020	2.99E-08	7.17E-12	1.26E-12
90	0.3	0.009	0.012	0.021	1.74E-08	3.80E-12	9.61E-13
90	0.4	0.009	0.013	0.023	7.90E-09	1.57E-11	3.27E-12
90	0.5	0.009	0.013	0.025	1.34E-08	3.68E-11	4.41E-12
90	0.7	0.008	0.014	0.027	6.14E-09	5.78E-11	8.35E-12
90	0.9	0.008	0.016	0.030	6.21E-09	1.90E-10	7.89E-11
90	0.999	0.008	0.016	0.031	3.72E-09	1.29E-10	5.54E-11

ST: Single trait GRS

MT: Multi-trait GRS [UKB info traits]

MT2: Multi-trait GRS [UKB info traits + T2DM]

* P-value comparing ST and MT

† P-value comparing ST and MT2

‡ P-value comparing MT and MT2

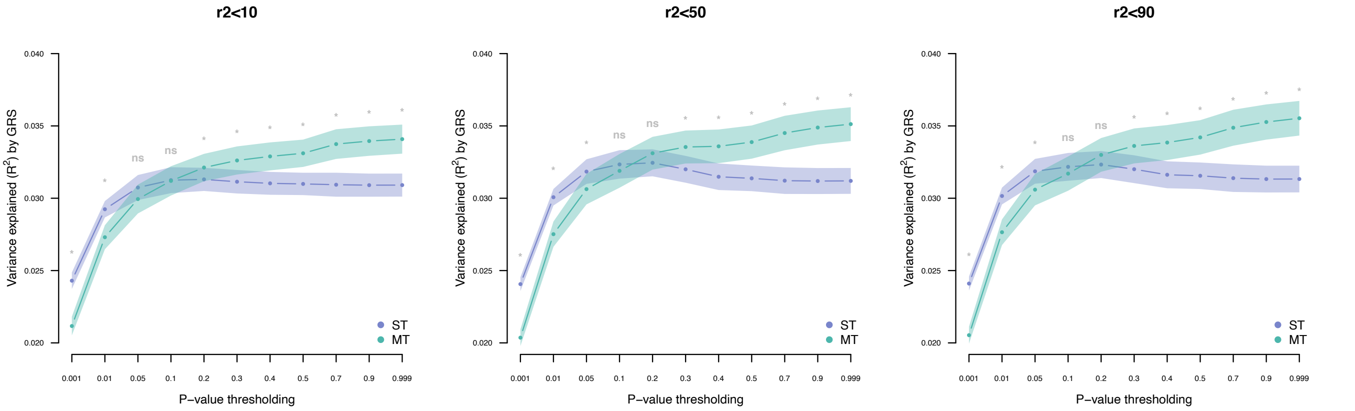

Figure S1 | Variance explained (R^2) for type 2 diabetes (T2D) by single-trait (ST) and multi-trait (MT) genetic risk scores by *P*-value thresholding and three levels of LD pruning levels. Left panel: remove variants with $r^2<0.1$, center panel: remove variants with $r^2<0.5$ and right panel: remove variants with $r^2<0.9$. Points indicate mean R^2 for a given threshold, and the surrounding shading indicates the standard error of the mean. ns: non-significant difference between ST and MT, *: significant difference between ST and MT.

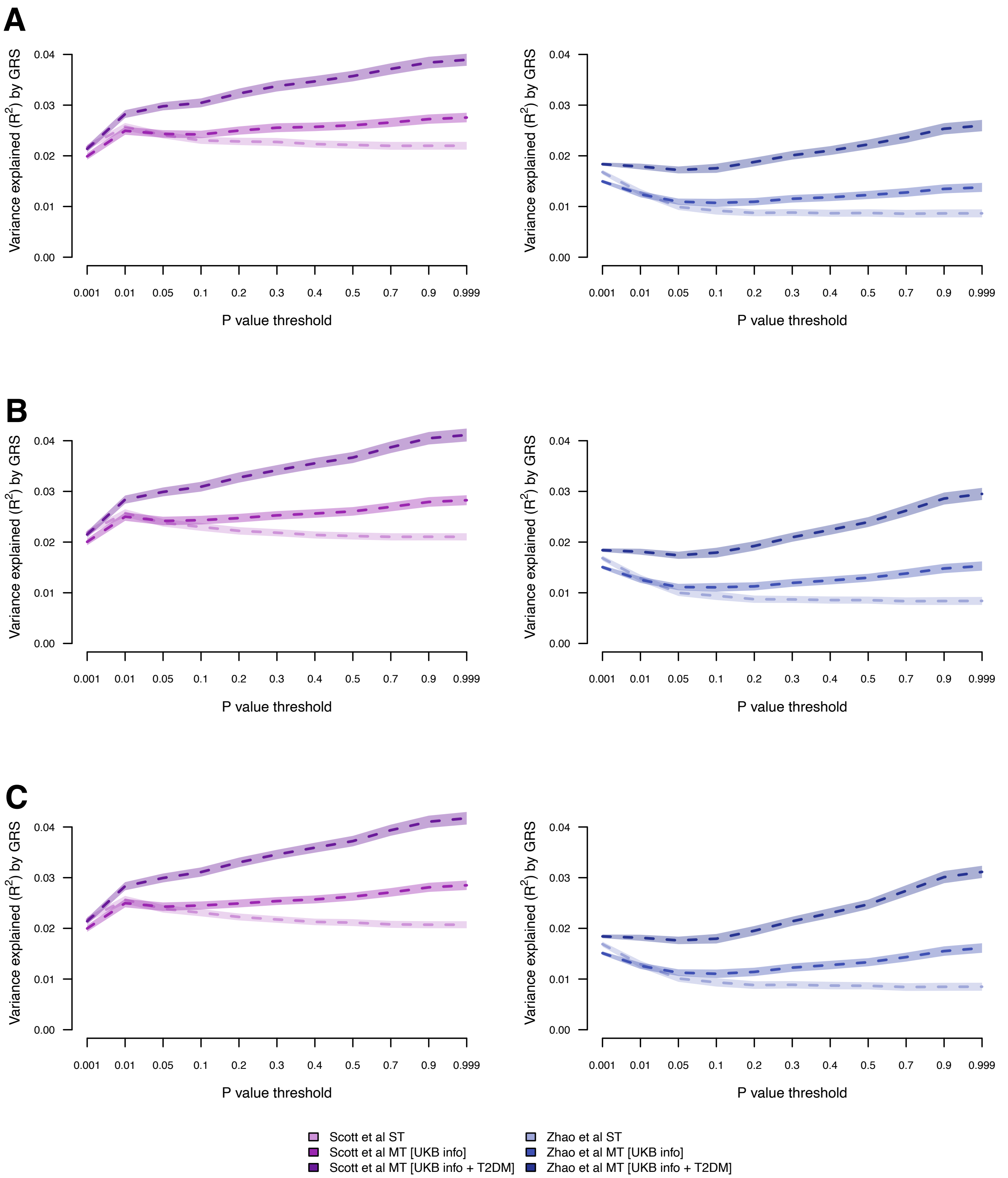

Figure S2 | Variance explained (R^2) for type 2 diabetes by single-trait (ST) and multi-trait (MT) genetic risk score for LD pruning $r^2<0.1$ [panel A], $r^2<0.5$ [panel B], $r^2<0.9$ [panel C]. The GRS was constructed based on publicly available summary statistics from Scott et al (2017) [left panel] and Zhao et al (2017) [right panel].

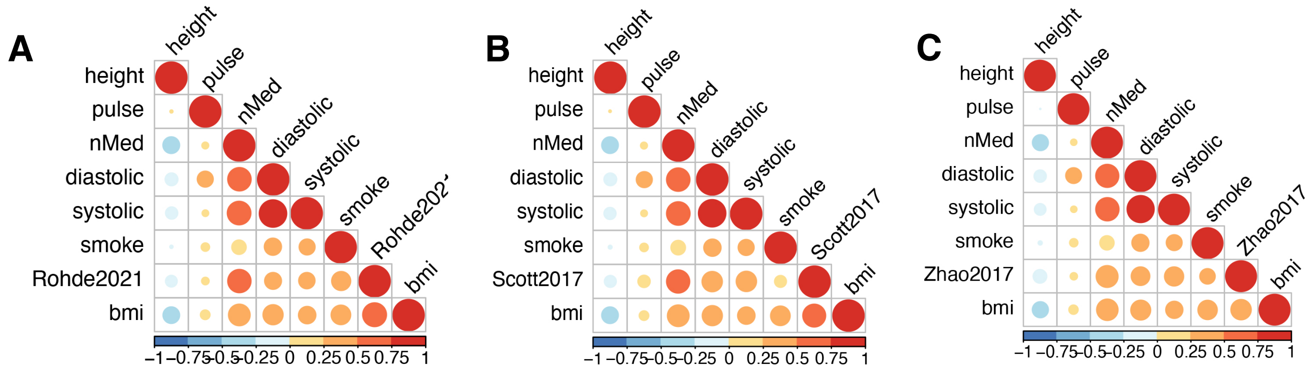

Figure S3 | Estimated genetic correlations among UKBB information traits and type 2 diabetes (T2D) obtained from **A**) UKBB [current study], **B**): Scott et al 2017 and **C**) Zhao et al 2017.

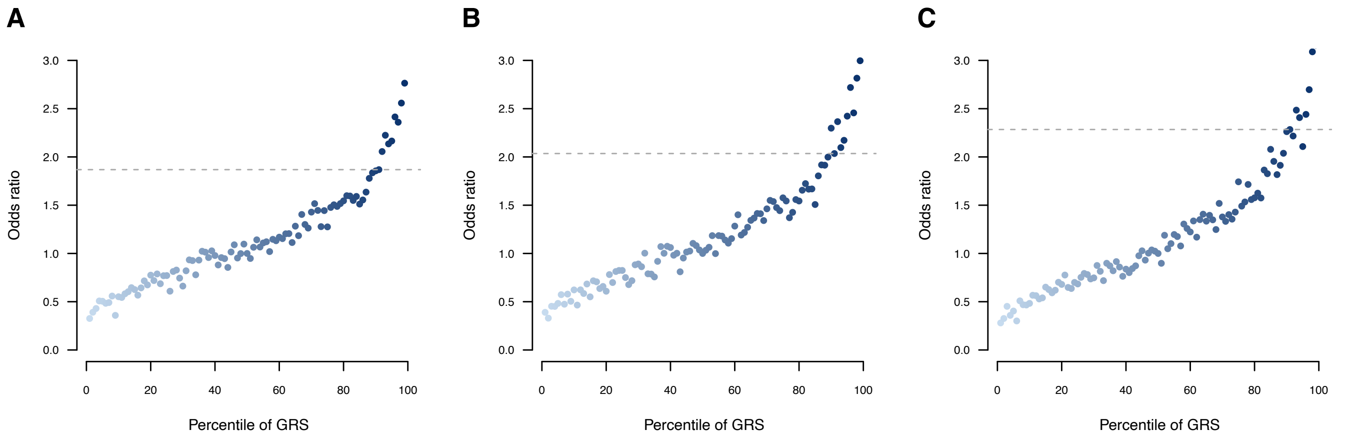

Figure S4 | Type 2 diabetes risk gradient according to genetic risk score percentile for **A**) the single-trait model, **B**) multi-trait model using the seven information traits, and **C**) multi-trait model with the seven information traits and T2DM summary statistics For each percentile the odds ratio was computed from logistic regression accounting for sex, age UKBB assessment center and first 10 genetic principal components. The reference point was set to percentile 50 (OR=1). Horizontal lines show the odds ratio of the 91st percentile.

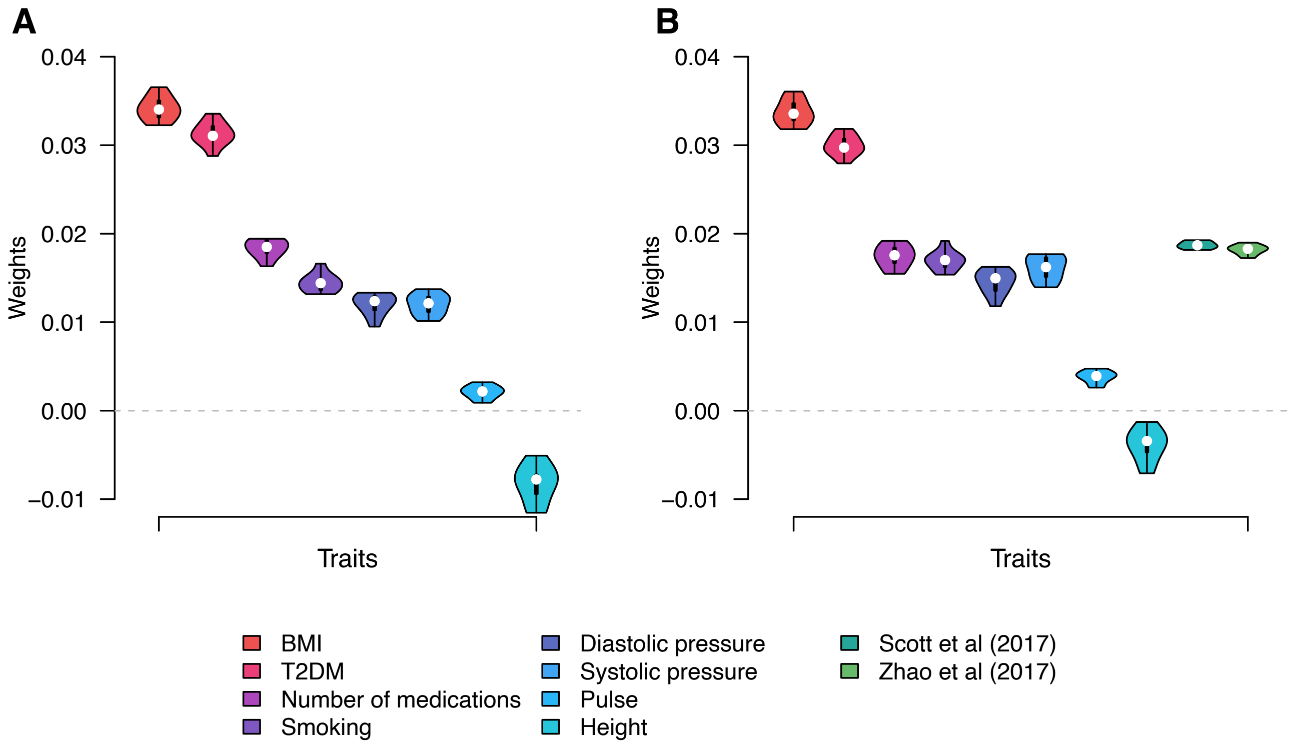

Figure S5 | Weights across the ten training sets for **A**) MT-GRS using the seven information traits, and **B**) MT-GRS using the seven information traits including two publicly available T2DM summary statistics.
